# Modeling of Knowledge Toward Herbal Medicine for Oral Health Using Multiple Linear Regression and Neural Network

**DOI:** 10.7759/cureus.41790

**Published:** 2023-07-12

**Authors:** Abedelmalek Tabnjh, Wan Muhamad Amir W Ahmad, Ruhaya Hasan

**Affiliations:** 1 Dental Sciences, Universiti Sains Malaysia, Kelantan, MYS; 2 Applied Dental Sciences, Jordan University of Science and Technology, Irbid, JOR

**Keywords:** knowledge, multiple linear regression, multilayer feedforward neural network, oral health, herbal medicine

## Abstract

Background and goals

Herbal medicine is used to treat a variety of oral health problems. Therefore, it is essential to comprehend it fully. To determine whether the amount used is risky, it is crucial to understand the dosages of medicinal plants. Before performing multiple linear regression (MLR) modeling, this paper uses the multilayer feedforward (MLFF) neural network (NN) technique to propose the variable selection. A data set with socio-demographic variables for dental staff and herbal medicine related to oral health knowledge score (KS) was chosen to demonstrate the design-build methodology.

Materials and methods

It was discovered that the KS is significantly related to the sex, age, income, occupation, and practice score (PS) at the first stage of the selection process, where all the variables were screened for their clinical importance. These five variables are chosen and used as inputs for the MLFF model by considering the level of significance, alpha = 0.05. Then, using the best variable discovered by the MLFF process, the MLR is applied.

Results

The performance of MLFF was evaluated using the mean squared error (MSE). MSE measures how far our estimates are off from the actual results. The MLFF’s smallest MSE indicates the model’s ideal combination of variable selection.

Conclusion

This study showed that using MLFF would help confirm the selected independent variables for MLR. In addition, KS level is more correlated with occupation, PS, and sex than with age and income. Moreover, this model could be used practically for any dataset. with the same criteria.

## Introduction

Many oral health issues like toothache, oral hygiene, bad breath, and fungal or bacterial inflammations, are treated with herbal medicine. Therefore, having a thorough understanding of it is crucial. Knowing the doses of medicinal plants is essential for figuring out whether the amount used is risky. Furthermore, safety awareness is crucial. All medicinal herbs and plants should be examined to determine whether they are suitable for treatment. The second type of information is the duration of herb therapy because prolonged use of some herbs may cause toxicity [1]. When using herbal treatments to treat various oral disorders, patients should know the reactions, benefits, and potential adverse effects [2]. Additionally, those who provide oral healthcare should collect data on the properties of herbs and plants that are ingested orally since they are a trustworthy provider of medical knowledge [2].

Even though many herbs are risk-free and without side effects, some have been shown to have adverse effects. Pulp necrosis was one of some herbs’ detrimental effects on the mouth, which relieved toothaches. Additionally, some herbs can result in burns, which are frequently linked to oral ulcers and persistent halitosis [3]. Deodorization with garlic extract, drinking dairy products, mushroom extract, tea catechins, plant extracts with polyphenol and phenolic derivatives, and honey are all traditional therapies for bad breath. Therefore, understanding herbs is crucial when treating oral diseases, especially when using unusual plants [3].

Regression analysis is frequently used in various research fields, including herbal medicine, to examine the link between knowledge level and practice and other variables. One of the most famous regression techniques is multiple linear regression (MLR). However, it is highly uncommon to discover research on practices and knowledge, including those on herbal medicine, that utilized bootstrapped data in a neural network (NN) model to examine the relationships between variables. As a result, bootstrapping and NNs are still far from this area of research. The bootstrap framework generates sample statistics by selecting a sample representative of the population. In order to generate a pseudo-population, bootstrap replicates the initial sample set multiple times and then makes a series of substitutions. Using the bootstrap method, generating a sample of the same size as the initial sample is possible. However, at this stage, some findings are repeated multiple times while others are eliminated. Different from samples obtained by chance alone are those obtained through random sampling and substitution. As replacement samples are chosen, the bootstrap method generates statistics for each sample [4].

Using NNs, we iteratively try to maximize output while minimizing error [5]. Recent research has focused on NN models to attain human-like performance across various knowledge engineering fields. Numerous fields are quickly adopting applications of NNs as tools for artificial intelligence. Implementing NNs has been tried by researchers for a variety of reasons [6]. Multilayer feedforward (MLFF) is one of the most commonly employed artificial NN techniques.

This study aims to improve model results through bootstrapping and ensure that the correct variables were picked as independent variables for the multilayer perceptron (MLP) model through MLFF. Moreover, this study aims to create a model to predict knowledge scores for using herbal products for oral health.

## Materials and methods

Data on the dental staff's knowledge of herbal medicine related to oral health was collected from the School of Dental Sciences, Universiti Sains Malaysia (USM), Kelantan, Malaysia. The study included 100 participants in total.

This is a cross-sectional study that is both retrospective and computational.

Participants

The study's reference population involved the dental team at USM's School of Dental Sciences. Respondents included lecturers, dental surgical assistants (DSA), nurses, office staff, and dental technicians. The inclusion criteria were Individuals with Malaysian citizenship, and ages 18 to 60 were chosen. As exclusion criteria, non-USM staff (e.g., cleaners) and those not Malaysians were excluded from this study.

Data collection and tools

In this study, a customized questionnaire that was suitable for oral health was used. It was adapted from a 2007 Azriani Bt Ab. Raman's study on the use of herbal remedies during pregnancy [7]. The practice score and socio-demographic variables served as independent variables, while the dependent variable was the knowledge score regarding herbal medicines. The questionnaire underwent a validation process. The procedure includes content validation with a group of experts, face validation with 10 Malay people, and a pilot study with 30 dental staff members.

There were five knowledge-based questions on herbal remedies for oral health included in the knowledge section. The questions concerned sources of information on herbs, familiarity with herbs used for oral health, names of well-known herbs, proficiency with herbs, and knowledge of effective ingredients. The practice section consisted of five questions. These included using herbal toothpaste, past use, the herbs themselves, how frequently they were consumed, and the reasons behind it.

For scoring purposes, a "1" mark was given for each accurate response in the knowledge domain, while a "0" mark was given for uncertain or do not know. For names of herbs, a mark of "1" was given for each recognized herb [8,9]. The knowledge section's overall score range was "0 to 10."

Multilayer feedforward neural network

The most well-known artificial NN, the multilayer feedforward neural networking (MLFFNN) method, was employed. The three main layers that make up MLFF are typically the input, hidden, and output layers [10-12]. Because there is only one dependent variable in the investigation study, there is only one output node for this analysis. The MLFF produced by the equation has N input nodes, H hidden nodes, and one output node. The following equation (1) is how the value is shown:

y ~ x1+x2+x3+…+ x(i-1) ……………1

**Figure 1:**
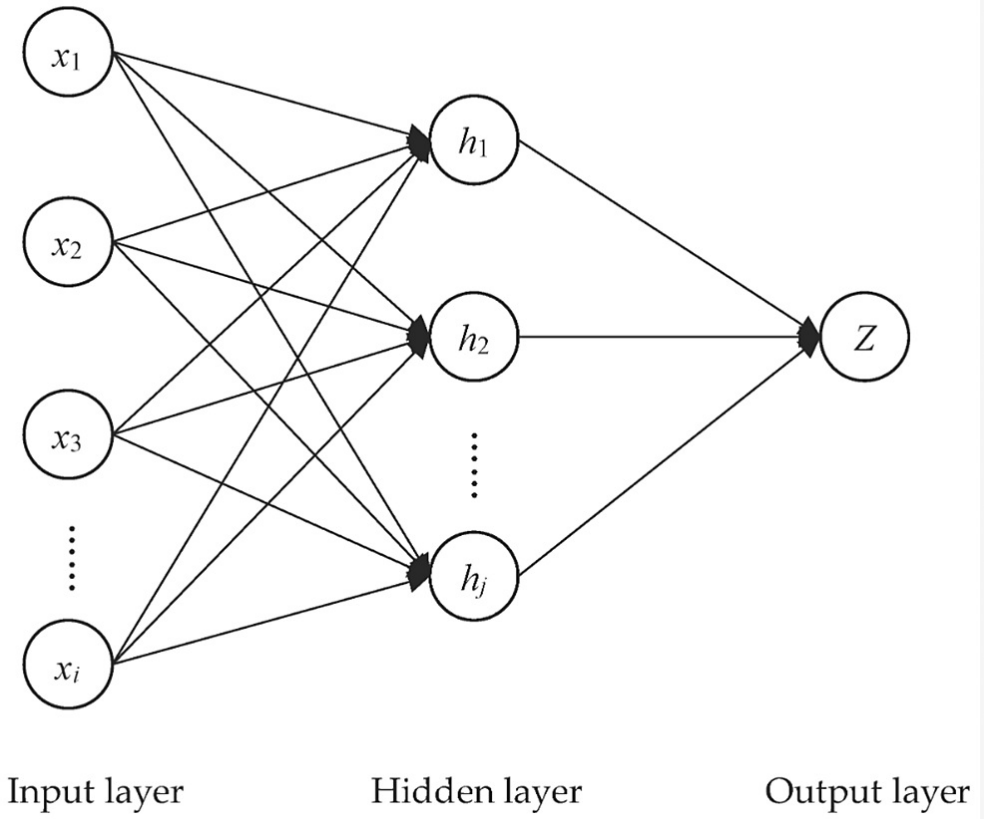
A MLP with one hidden layer, (i) input nodes, (h) hidden nodes, and one output node x: variables, i: number of input nodes, h: hidden nodes, j: number of hidden nodes, Z: output node

Figure 1 illustrates the overall architecture of the MLP model. The MLR procedures will input the chosen variable from the MLFF procedure [13]. These are the details of the suggested model:

KS = Sex + Age + Income + Lecturers + Nurses + DSA + Technicians + PS

As independent variables, practice score (PS), sex, age, income, and occupation were chosen based on the literature. The occupation variable was transformed into binary variables to meet linear regression assumptions. The administrator workers group was chosen as the reference group.

Data analysis

The syntax has been written entirely in the R programming language for statistical analysis: the MLR modeling with embedded bootstrapping and MLFF syntax.

Eight variables have been chosen in this case as shown in Table 1, and they define X1 (sex), X2 (age), X3 (income), X4 (lecturer), X5 ( nurses), X6 (DSA), X7 (technicians), and X8 (PS). MLFF was used to test all specified variables, and the most significant variable was used for regression modeling. The present study split the dataset into a training set of 70% and a testing set of 30%. One hidden layer MLFF is the most suitable model for the examined case.

**Table 1:** Description of the study's chosen variable's data Y: dependent variables, X: independent variables, KS: knowledge score, DSA: dental surgical assistant, PS: practice score

n	Variables-code	Explanation of variables
1	Y	KS
2	X1	Sex
3	X2	Age
4	X3	Income
5	X4	Lecturers
6	X5	Nurses
7	X6	DSA
8	X7	Technicians
9	X8	PS

## Results

**Figure 2:**
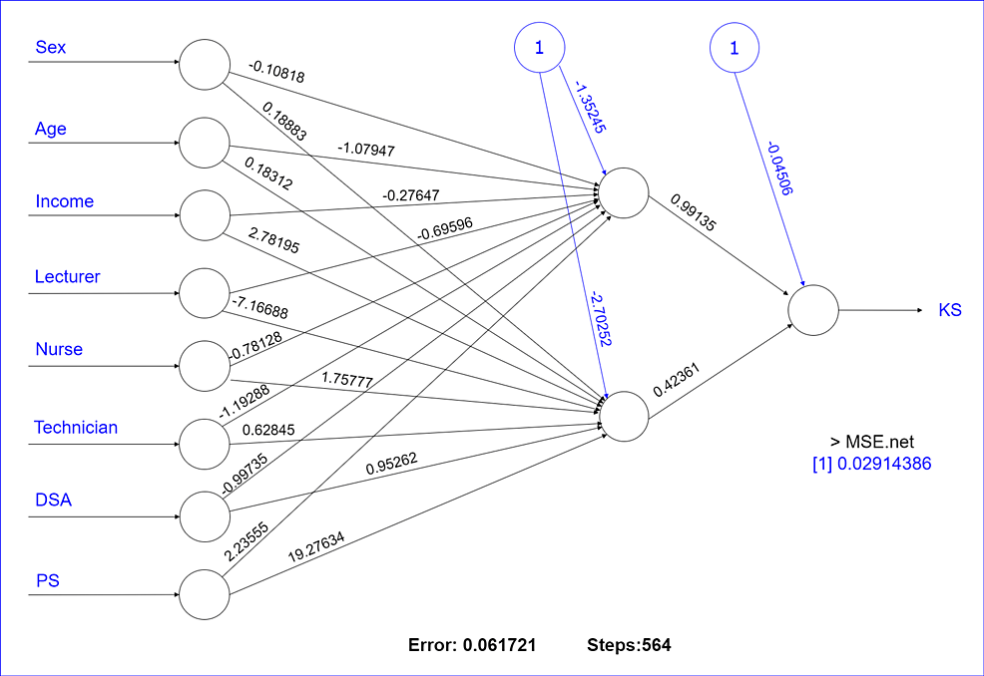
The MLFF architecture with eight input nodes, one concealed layer containing two neurons, and one output node MSE: mean squared error, PS: practice score, KS: knowledge score

Figure 2 illustrates the architecture of the MLFF, which consists of eight input nodes, a hidden layer containing two neurons, and one output node. The variables in this section were identified using the established MLFF methodology. Sex, age, income, occupation, and PS are the five factors that have had the most significant impact on the knowledge score (KS). The current study aims to examine how the MLFFNN and MLR perform. The best MLFF model was determined by which combination of the selected variables produces the minimum mean squared error (MSE). All MSEs from the run MLFF were listed and taken to get this result, each with a different set of variables.

The variables that (based on the smallest MSE) have the most significant impact on the KS were used as independent variables for the MLR. The MLFF proposed in this study has one output, one hidden layer, and eight input nodes. In this research, the output node (KS, a dependent variable) was set to one. According to the 70:30 train-to-test divide, 70% of the data were utilized for network training, while the remaining 30% is available for network testing [10,11,14]. The testing/out-sample MSE was used to evaluate the performance of the MLFF. MSE demonstrates how far off from the actual results our estimates are. Table 2 provides a summary of the results of the multiple regression modeling.

**Table 2:** Coefficients result of multiple regression SE: standard error

	Estimate	SE	t-value	P-value
(Intercept)	5.30	2.27E-01	23.32	< 2e-16 ***
Sex	0.432	0.0608	7.098	2.40e-12 ***
Age	0.112	0.00706	-15.87	< 2e-16 ***
Income	0.000136	0.0000238	5.716	1.44e-08 ***
Lecturers	1.03	0.25	-4.127	3.99e-05 ***
Nurse	0.107	0.103	-1.04	0.298
Technicians	1.68	0.0846	-19.879	< 2e-16 ***
DSA	0.57	0.0784	-7.268	7.39e-13 ***
PS	0.633	0.00502	126.137	< 2e-16 ***

Consequently, the suggested linear model is provided by

KS = 5.298 + 0.432 (Sex) - 0.112 (Age) + 0.0001 (Income) - 1.030 (Lecturers) - 0.107 (Nurses) -1.682 (Technicians) - 0.570 (DSA) + 0.633(PS) ................... 2

Equation 2 represents the multiple linear models of the KS. The sex (b1: 0.432; P < 0.05) shows a significant relationship toward the KS. Age (b2: -0.112; P < 0.05) also shows a significant relationship to the level of KS. The third variable is income (b3: 0.0001; P < 0.05). Regarding occupation variables, all of them show a significant relationship toward KS (P < 0.05), except nurses (P > 0.05). The values were for lecturers (b4: -1.030), nurses (b5: -0.107), technicians (b6: -1.68), and DSA (b7: -0.570). PS also showed a significant relationship with KS (b8: 0.633; P < 0.05).

## Discussion

The primary focus of this study is the creation of methodologies for MLFF and MLR. Data were first bootstrapped to improve accuracy before being split into a training dataset (70%) and a testing dataset (30%). At one hidden layer, the MLFF model was used. In this study, we determine the MLFF and MLR mean MSE. This can also be used as a method of variable selection. Its purpose is to evaluate the network's performance. It was decided to use the MLFF model with the smallest MSE. The MLR model was built using the input from the MLFF. Doing this will ensure that the model obtained is the best model for the intended prediction.

According to the MLR equation, sex, age, and income, all have a significant minimal effect on KS. This may help to explain why some studies find links between these characteristics and KS, whereas other studies find none. For instance, Farooqui et al. (2016) discovered a link between KS and sex [15]. However, Tubaishat et al. (2005) found no relationship between age and sex when utilizing medicinal herbs for oral health [16]. Because of this discrepancy in the literature, there is not a significant association between these socio-demographic factors on the one hand and KS on the other. As a result, sociodemographics have a negligible impact on the KS score, as demonstrated by the MLR findings in this study. This study demonstrated a significant effect on the link between KS and occupations, consistent with Farooque et al. (2016).

Numerous research contends that the two variables have a significant connection when examining the relationship between KS and PS. In a study conducted by a dental hygienist in California, Hurlbutt et al. (2011) found a significant association between KS and PS regarding herbal medicine [17]. A significant association between oral health knowledge and practice was also demonstrated by Lin et al. in (2001) [18].

For further development, some restrictions should be taken into consideration. The results of this investigation should be interpreted with consideration of its context. This study is limited to dealing with MLR and the MLFFNN. As a result, this study did not analyze other aspects of herbal medicine, such as attitudes, which were primarily concerned with creating the technique. Second, the data used in this study were restricted to knowledge and practice of herbal medicine associated with oral health.

## Conclusions

This study showed that using MLFF would help confirm the selected independent variables for MLR. In addition, these five factors, sex, age, income, occupation, and PS, would enhance the KS. Occupation, PS, and sex correlate more with KS level than age and income. The methods presented in this thesis's result can be used to create regression models that are very precise and validated using the herbal medicine related to oral health KS dataset, as well as other datasets that satisfy the established assumptions.
